# Tobacco-Free Nicotine Pouches and Their Potential Contribution to Tobacco Harm Reduction: A Scoping Review

**DOI:** 10.7759/cureus.54228

**Published:** 2024-02-15

**Authors:** Erika Grandolfo, Henry Ogden, Ian M Fearon, Layla Malt, Matthew Stevenson, Sarah Weaver, Thomas Nahde

**Affiliations:** 1 Group Science and Regulatory Affairs, Imperial Brands PLC, Bristol, GBR; 2 Scientific Research, whatIF? Consulting Ltd., Harwell, GBR; 3 Group Science and Regulatory Affairs, Reemtsma Cigarettenfabriken, Hamburg, DEU

**Keywords:** tobacco-free nicotine pouches, scoping review, tobacco harm reduction, cigarette smoking, oral nicotine delivery systems

## Abstract

Tobacco harm reduction (THR) refers to strategies designed to reduce the health risks associated with tobacco smoking but may involve continued use of nicotine and/or tobacco. Next-generation products (NGPs) are a THR alternative as they do not burn tobacco or produce smoke and deliver nicotine and have fewer and substantially lower levels of harmful chemicals compared to cigarettes. Tobacco‑free nicotine pouches (TFNPs) are an emerging category of nicotine‑containing oral products that do not combust or contain tobacco leaf. Similar to Swedish snus, TFNPs are placed between a user’s lip and gum, and nicotine is absorbed through the oral mucosa rather than being inhaled. The aim of this scoping review was to systematically collate and evaluate published scientific evidence (cut‑off of 31 May 2023) identified from bibliometric databases investigating the potential of TFNPs to contribute to THR. Overall, studies examining chemical constituents indicated that the use of TFNPs may result in lower exposure to toxicants than other tobacco or nicotine-containing products, both combustible and non‑combustible. This reduction in toxicant exposure has been demonstrated by multiple human biomarker studies and *in vitro* toxicological assessments to translate to harm reduction potential in smokers switching to TFNPs. However, further study is warranted. At present, there is some evidence from human behavioral research that TFNPs can support either transitioning away from smoking or reducing cigarette consumption. Furthermore, TFNP use appears very much limited to current users of traditional tobacco products, and youth uptake has been limited. In conclusion, the findings of this review indicate that TFNPs have the potential to support THR efforts and may help inform evidence‑based regulation.

## Introduction and background

Cigarette smoking is a cause of serious diseases in smokers, including lung cancer, heart disease, and emphysema. Smoking reportedly causes more than eight million deaths per year globally [1], and, in the United States (US), almost 500,000 annual deaths are reported to be attributed to cigarette smoking [2]. The greatest risk of smoking-related disease comes from burning tobacco and inhaling smoke. Combusting (burning) tobacco produces smoke containing around 7,000 chemicals, of which around 100 are classified by public health experts as causes or potential causes of smoking-related diseases [3]. These public health experts widely conclude that, while nicotine is addictive and not risk-free, it is the toxicants in cigarette smoke generated by burning tobacco and not the nicotine that causes smoking-related diseases [4,5].

The best action adult smokers can take to improve their health is to stop smoking [6]. In the US, a large number of adult smokers report wanting or attempting to transition away from cigarettes [7,8]; however, only a small proportion stop smoking each year [8]. Despite the known risks of smoking, many adult smokers are uninterested or unwilling to stop smoking [9,10]. Tobacco harm reduction (THR) refers to strategies designed to reduce the health risks associated with tobacco smoking, which may involve continued use of nicotine and/or tobacco [11,12]. In recent years, product categories have emerged that deliver nicotine without burning tobacco. Next-generation products (NGPs), such as electronic vapor products (EVPs), heated tobacco products (HTPs), and tobacco-free oral nicotine pouches (TFNPs), eliminate the process of tobacco combustion, meaning they contain and produce fewer and significantly lower levels of harmful chemicals compared to cigarette smoke. A growing number of public health bodies have proposed that novel products that deliver nicotine without burning tobacco may provide less harmful alternatives to cigarettes [5,7,13-16].

In consideration of different categories of nicotine-containing products, these do not all carry the same level of risk. Science demonstrates that the way in which nicotine is delivered to the consumer (i.e., the delivery mechanism) plays an important role in determining the level of risk associated with using a particular product. While burning tobacco to release nicotine and inhaling the smoke is the most crucial element determining a product’s risk profile, consideration must also be given to whether products also contain tobacco leaf (even if it is not combusted/burnt) and whether the nicotine is delivered via inhalation to the lungs or some other methods.

**Figure 1:**
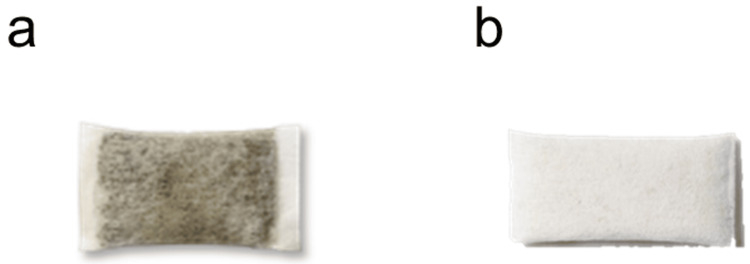
Swedish Snus and a Tobacco-Free Nicotine Pouch. Illustration comparing (a) a tobacco containing Swedish snus (Skruf, Imperial Brands) with (b) a tobacco-free nicotine pouch (ZoneX, Imperial Brands). Source: Imperial Brands PLC

Oral nicotine delivery systems (ONDS), such as snus (a moist oral tobacco product that is placed behind the upper lip, either loose or in portioned sachets), are one product category that allows nicotine consumption without tobacco combustion and have been widely reported as potentially reduced-risk products in comparison to cigarettes [17,18]. Recently, TFNPs have emerged as a new category of ONDS products, which contain nicotine, but, unlike snus, do not contain tobacco [19] (Figure 1). Typically, TFNPs contain pharmaceutical‑grade high-purity nicotine, either derived from tobacco or synthetic, that is either combined with a plant fibre‑based substrate (e.g., wheat or bamboo) or in a dry powder format. Other ingredients, such as flavourings, humectants, and additives, are added to aid processing and to help maintain product stability. Like traditional snus, TFNPs are placed between a user’s lip and gum for a certain period of time, steadily releasing nicotine, which is absorbed into the bloodstream through the oral mucosa. As TFNPs do not contain tobacco leaf, related toxicants that naturally occur in both combusted and non‑combusted tobacco, such as tobacco‑specific nitrosamines (TSNAs), are likely to be substantially reduced. Consequently, encouraging either smokers or snus users to switch to TFNPs could represent a significant opportunity for THR at both the individual and population levels [20].

In recent years, various brands of TFNPs have been developed and marketed in many countries, which offer a variety of different flavours, nicotine contents and free nicotine levels, moisture and pH levels, and formats [21]. Since TFNPs are a relatively new category of products, long‑term epidemiology data on the potential health effects associated with their use are not yet available. Due to this, some regulators are relatively cautious about the introduction of these products into consumer markets [22]. A successful THR approach requires proportionate regulation that acknowledges both the potentially reduced relative risk profile of TFNPs compared to products that contain tobacco, especially cigarettes, and the support that TFNPs may provide to adult smokers wanting to switch away from cigarette smoking. Proportionate regulation should ensure that TFNPs reduce smoking prevalence and smoking‑related harms without increasing the initiation of nicotine consumption among unintended users. At present, TFNP regulations differ markedly around the world. In the United Kingdom (UK) and the European Union (EU), TFNPs are regulated under the Classification, Labelling and Packaging (CLP) regulation. Some EU markets have also introduced specific TFNP regulations, for example by implementing age restrictions (Sweden), requiring sales permits to be issued to manufacturers and regulating pouch nicotine content (Finland), or banning the sale of TFNPs outside of specialist tobacco stores (Czech Republic). In the US, TFNPs fall under the oversight of the Food and Drug Administration (FDA), and pre‑market authorisation for TFNPs is required via the pre‑market tobacco product application (PMTA) program. In contrast, in countries such as Australia, Brunei, Iran, Mauritius, New Zealand, and Singapore, TFNPs are banned unless they are approved as medicines [23].

Given the perceived relative lack of awareness of TFNPs and their potential to make a meaningful contribution to THR, the aim of this review is to collate and evaluate the available scientific evidence. This includes an assessment of any individual health impacts, in addition to population‑level behavioural effects, such as helping smokers switch and initiation of use among nicotine non‑users. The findings of this review intend to provide greater awareness of scientific evidence available on TFNPs to date, as well as informed discussion about the THR potential of TFNPs, and may help inform evidence‑based regulation.

## Review

Methods

Information Sources and Search Terms

To identify relevant literature, the bibliometric databases PubMed and the Cochrane Central Register of Controlled Trials (CENTRAL) were searched from inception to May 31, 2023. The following search terms were applied: “tobacco‑free nicotine [Title/Abstract]” OR “oral nicotine [Title/Abstract]” OR “nicotine pouch [Title/Abstract]” OR “nicotine pouches [Title/Abstract]”. All searches were conducted by a single author. Additional papers were also retrieved from the reference lists of included literature and from the authors’ independent literature repositories.

Study Eligibility Criteria

Studies were included if they examined the impact of TFNPs in the context of THR either alone or compared with cigarette smoking; NGP use (e.g., HTPs, snus, EVPs); nicotine replacement therapy (NRT) use, including dermal patches, nicotine gum, nicotine lozenges, and nicotine sprays; or no tobacco/nicotine product use. Peer-reviewed original research was included if it was published in the English language prior to the search cut‑off date. Articles that had not been peer‑reviewed, including clinical trial pre‑registrations, study pre‑prints, conference proceedings, media articles, book chapters, and internal industry reports, were all excluded. Similarly, non‑original research, including narrative reviews, systematic reviews, meta‑analyses, editorials, opinion papers, and letters to the editor, were all excluded. Any outcome measure with relevance to the meaningful impact of TFNPs in the context of THR was considered relevant for inclusion, except papers relating to product regulation and industry marketing, which did not present original data. No restrictions were applied for specific TFNP products; however, studies examining individual ingredients in isolation, which are found within TFNPs (e.g., nicotine, flavourings), were not considered relevant.

Selection of Evidence Sources 

Search results were imported into Rayyan (Rayyan Systems Inc., Cambridge, MA) systematic review software [24]. Duplicate records were screened and removed automatically in Rayyan. Title and abstract screening were conducted independently by two authors, in which studies were classified in terms of relevance as “yes”, “no” or “maybe”. Each study required two “yes” classifications to move forward to the full-text screening phase. Any disagreements in study classification during the title and abstract screening were resolved by discussion before proceeding. Full‑text screening was also conducted by two authors with studies being classified as “yes” or “no” for inclusion.

Critical Appraisal

This scoping review was drafted in accordance with the guidelines proposed by the Preferred Reporting Items for Systematic Reviews and Meta-Analyses Extension for Scoping Reviews (PRISMA-ScR) checklist [25]. The evidence-based reporting system for systematic reviews set forth by the PRISMA group advises that conducting a formal assessment of an individual study's methodological quality is not a typical feature of a scoping review [25]. As such, no formal assessment of quality was included in this paper.

Results

**Figure 2:**
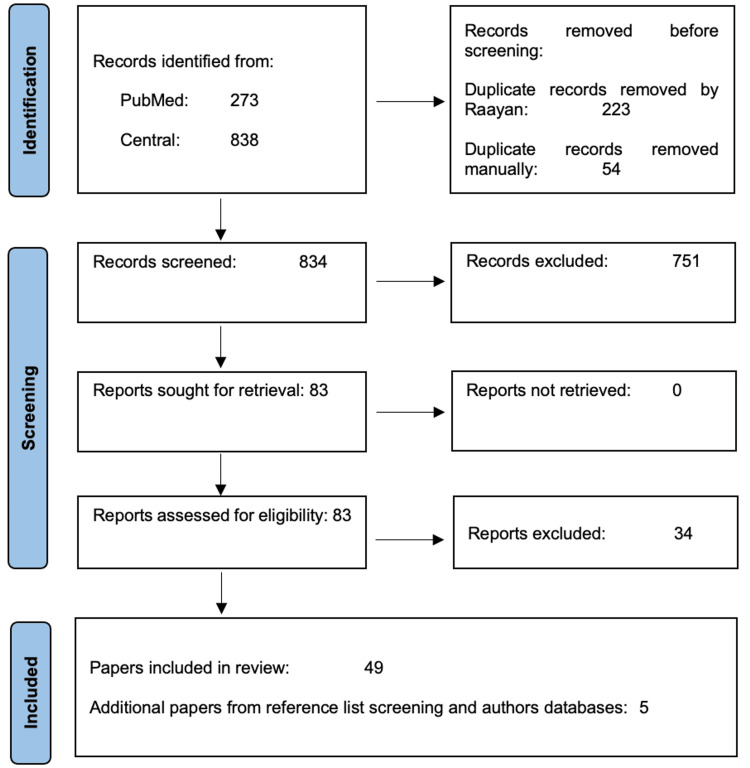
PRISMA Flow Diagram Depicting the Process of Paper Selection.

Information detailing the number of articles identified, screened, and discussed in this review, can be found in Figure 2. Database searches identified 1,111 potentially eligible published articles. Following title and abstract screening, and assessment of eligibility, a total of 49 articles were deemed eligible for inclusion. A further five articles were identified from manual reference list searches of identified articles and through the authors’ personal databases. Of these 54 papers, 11 were related to chemical constituents and toxicological impacts; eight were related to nicotine pharmacokinetics; six were related to biomarkers and disease/health endpoints related to TFNP use; 11 were related to prevalence and motivation to use in youth; 14 were related to prevalence of use, intentions to use, and actual use in adults (noting that two of the papers identified related to both youth and adult TFNP use [26,27]), four were related to emerging topics on perceptions, including the use of "tobacco‑free" terminology in branding and marketing; and two were related to TFNP sales data.

Chemical Analysis of TFNPs

Public health bodies, including the Royal College of Physicians and FDA, state that, while nicotine is addictive and not risk-free, it is the toxicants in cigarette smoke generated by burning tobacco, and not nicotine, that cause smoking-related diseases [4,5]. When assessing the harm reduction potential of NGPs, an important first step is to evaluate the number and amount of potentially harmful toxicants in TFNPs relative to cigarette smoke. Four studies were identified that examined such chemical constituents of TFNPs.

In an analysis of the presence of 26 analytes relevant to oral tobacco products, Azzopardi et al. [28] assessed harmful and potentially harmful constituents (HPHCs) from the FDA smokeless tobacco reporting list [3], the GOTHIATEK® Standard list of toxicants [29], and nine smoke constituents prioritised by the World Health Organization’s Tobacco Product Regulation Group (TobReg) [30] in extracts from various TFNPs and two commercially available NRTs (Nicorette lozenge and gum). For the TFNPs, in addition to moisture, only three of the 26 measured compounds were present at quantifiable levels, and these included nicotine. Two toxicants detected in the TFNPs, chromium and formaldehyde, were present at extremely low levels, close to the quantification limits. For the nicotine lozenge, only nickel and chromium were reported at quantifiable levels. Similarly, for the nicotine gum, only low levels of cadmium, chromium, nickel, and lead were detected. In the snus samples, the authors report that 11 toxicants were at quantifiable levels (namely, the heavy metals cadmium, chromium, nickel, arsenic, and lead; the TSNAs N-nitrosonornicotine (NNN), N-nitrosamines (NNK), and N-Nitrosodimethylamine (NDMA); the carbonyls formaldehyde and acetaldehyde; and one mycotoxin, ochratoxin A). The concentrations of these quantified toxicants in snus were all reportedly present at substantially higher levels than those in TFNPs or NRT. Azzopardi et al. [28] also utilised average daily consumption data taken from market surveys to compute exposure estimates for both snus and TFNPs. The authors reported that, across the product categories, TFNPs and NRT had the lowest toxicant profiles and estimations of relative toxicant exposure. Thus, the use of TFNPs appears to expose consumers to lower levels of toxic compounds than Swedish snus.

Jablonski et al. [31] assessed the levels of selected HPHCs in extracts from 25 ONDS products, which included 13 TFNPs manufactured using white granular powder, eight TFNPs manufactured using non‑tobacco plant material with nicotine added, and four smokeless tobacco products (two commercial products and two CORESTA smokeless tobacco reference products (CRP)). Levels of benzo[a]pyrene, nitrite, and TSNAs were below the limit of detection for 20, 14, and 19 of the 21 assessed TFNPs, respectively. With respect to carbonyls, levels of formaldehyde in TFNPs were reportedly comparable to those present in smokeless tobacco products. Acetaldehyde levels were reported below the limit of detection or limit of quantification for those TFNPs in the dry powder format, while levels in plant‑based products were reportedly considerably more variable and in some cases equivalent to those found in smokeless tobacco products. Crotonaldehyde was not detected in 20 of the 21 TFNPs or in any of the smokeless tobacco products. For the metals, the authors reported levels of arsenic, beryllium, cadmium, and selenium below the limit of quantification in the dry powder TFNP, while plant‑based TFNPs contained quantifiable but low levels of each metal. Similar to the findings of Azzopardi et al. [28], based on levels of measured toxicants, data from Jablonski et al. [31] suggest that TFNPs likely pose a much‑reduced exposure risk compared with smokeless tobacco products. Additionally, these findings also suggest that dry powder-based TFNPs displayed lower levels of the HPHCs assessed compared to plant‑based TFNPs, and this trend was most evident in the metals and acetaldehyde analysis. According to the authors, this was likely due to a combination of higher native levels in the plants being used and any curing or manufacturing processes employed during production.

Adopting a similar study design to Azzopardi et al. [28], Back et al. [32] examined the presence of 43 compounds in extracts from various ONDS, including dry and moist varieties of the "ZYN" TFNP, "General Snus", the CRP2.1 reference smokeless tobacco product, and "Grizzly Pouches Wintergreen" moist snuff products. Pharmaceutical nicotine lozenge and gum NRT were also assessed. The compounds tested included 43 chemicals, 36 of which are classified as HPHCs by the US FDA [3], and 16 compounds from the GOTHIATEK® Standard list of toxicants [29]. Eleven of them are on the FDA HPHC list, of which nine are relevant for smokeless tobacco products. Tested compounds also included four TSNAs, two of which appear on both the FDA HPHC and GOTHIATEK® lists. In this analysis, for both ZYN TFNPs, 28 of the 43 analysed compounds were below their respective levels of quantification. These included TSNAs and polyaromatic hydrocarbons. Formaldehyde, chromium, and ammonia were reported at low levels in both ZYN variants. Formaldehyde and chromium were reportedly slightly higher in the dry ZYN TFNP compared with the moist ZYN TFNP. Trace levels of nickel were reported in the dry ZYN TFNP; however, levels were lower than those measured for both the pharmaceutical nicotine lozenge and gum. Chromium was detected in the NRT gum, at approximately 5‑fold higher levels than in the dry ZYN TFNP. All compounds quantified in the ZYN products were also detected in snus and moist snuff products, but the levels were substantially lower in both ZYN TFNP. In addition, snus extracts contained chemicals not detected in the TFNP extracts, such as TSNA, acetaldehyde, and ochratoxin A. Overall, data from Back et al. [32] support the findings of Azzopardi et al. [28] in that, of the products tested, the relative exposure profile to many toxicants is likely highest for snus and lower and similar for TFNPs and NRT, respectively (Table 1).

**Table 1:** Example Data Concerning Potential Toxicant Levels in TFNP Products. Levels of potential toxicants reported in studies on chemical constituents of TFNPs from the references indicated. BLOQ, below the limit of detection or quantification; BLOQ*, below the limit of detection or quantification only for plant-based TFNPs; Q, quantifiable levels but lower than smokeless tobacco products; Q*, quantifiable levels comparable to smokeless tobacco products; -, not tested

Analyte	Reference		
	Azzopardi et al. [28]	Jablonski et al. [31]	Back et al. [32]
Formaldehyde	Q*	Q*	Q*
Acetaldehyde	BLOQ	BLOQ*	BLOQ
Acrolein	BLOQ	-	-
Crotonaldehyde	BLOQ	BLOQ	BLOQ
NAB	BLOQ	BLOQ	BLOQ
NAT	BLOQ	BLOQ	BLOQ
NNN	BLOQ	BLOQ	BLOQ
NNK	BLOQ	BLOQ	BLOQ
NDMA	BLOQ	-	BLOQ
Benzo(a)pyrene	BLOQ	BLOQ	BLOQ
Cadmium	BLOQ	BLOQ*	BLOQ
Chromium	Q	Q	Q
Mercury	BLOQ	-	BLOQ
Beryllium	-	BLOQ*	BLOQ
Selenium	-	BLOQ*	BLOQ
Nickel	BLOQ	Q	BLOQ*
Arsenic	BLOQ	BLOQ*	BLOQ
Lead	BLOQ	Q	BLOQ
Nitrite	BLOQ	BLOQ	BLOQ
Aflatoxin B1	BLOQ	-	BLOQ
Aflatoxin B2	BLOQ	-	BLOQ
Aflatoxin G1	BLOQ	-	BLOQ
Aflatoxin G2	BLOQ	-	BLOQ
Ochratoxin A	BLOQ	-	BLOQ

Mallock et al. [33] examined concentrations of total nicotine, freebase nicotine, and TSNAs in 44 TFNP variants, in addition to two variants of nicotine‑free pouches. All TFNPs were commercially purchased in Germany in 2021, and this study did not include any comparator or reference products within the analysis. Total nicotine content ranged from 1.79 to 47.5 mg per pouch (median 9.48 mg per pouch), with the median proportion of freebase nicotine being 86% (interquartile range: 62%-98%). The authors reported that the total nicotine concentrations were generally higher than what had been published previously for TFNPs by other authors, which was suggested to be potentially attributable to the inclusion of products sold by smaller brands within the analysis. TSNAs were detected in approximately half of the products tested, including quantifiable levels of NNN, NNK, NAT, and NAB in 15, three, two, and three products, respectively. Contextually, it should be highlighted, however, that, in accordance with studies by Azzopardi et al. [28], Jablonski et al. [31], and Back et al. [32], in which TSNAs were detected, these were only in trace concentrations (<13 ng per pouch, with pouch weights ranging from approximately 0.3 g to 1.2 g) [33], and substantially below the levels reported for smokeless tobacco products such as snus.

Toxicological Analysis of TFNPs

Toxicological assessments, including the use of validated *in vitro* models, are used to explore whether NGPs produce any biological responses in cells and can provide important insight into whether TFNPs have harm reduction potential relative to cigarettes. Six studies were identified that examined the toxicological impact of TFNPs, which are discussed below.

Bishop et al. [34] analysed the *in vitro* toxicological impact of a "Lyft" TFNP (4 mg, Berry Frost) with both CORESTA CRP1.1 reference 8 mg Swedish‑style snus [35] and 1R6F reference cigarettes [36]. The authors examined various endpoints, including cell viability, cell permeability, oxidative stress, and genotoxicity, in both human gingival fibroblasts (HGF) and human bronchial epithelial (H292) cells. Measurements were made after 24 hours of exposure to the test and reference samples. The observed cytotoxicity data suggested that the CRP1.1 smokeless (snus) tobacco product and the Lyft TFNP exhibited greatly reduced toxicity compared with 1R6F reference cigarette smoke extracts. In addition, CRP1.1 exhibited a greater toxicological response than Lyft TFNP extracts, which displayed minimal cytotoxicity and a slight reduction in cellular glutathione content (an indicator of oxidative stress). When taken together with the findings reported by Azzopardi et al. [28] and Jablonski et al. [31], these data substantiate the reduced‑risk potential of TFNPs compared with both Swedish snus and cigarettes.

Using real‑time cell analysis in HGF and human lung carcinoma (H292) cells, East et al. [37] examined the cytotoxic potential of nine non‑commercial Lyft TFNPs with different nicotine strengths and flavours, two commercial "Nordic Spirit" TFNP, and CORESTA CRP1.1 reference Swedish‑style snus. Both the HGF and the H292 cell lines exhibited approximately a 10-20% reduction in cell viability following a 24‑hour exposure period with extracts from three different Lyft flavours (Berry Frost 4 mg nicotine, Polar Mint 4 mg nicotine, and Tropical Breeze 6 mg nicotine), and reductions in viability were comparable to those seen with CRP1.1 snus extracts. Similar to the study by Bishop et al. [34], the authors reported that increasing nicotine strength did not significantly impact the cytotoxicity profile [37]. Overall, data arising from this study suggest that, at comparable extract concentrations, Lyft TFNP variants were less cytotoxic than extracts of the Nordic Spirit TFNP and the snus reference product.

Yu et al. [38] compared the biological activity of extracts obtained from two unnamed commercially available TFNPs with extracts from a snus product and total particulate matter (TPM, generated by capturing cigarette smoke particulate on a filter pad and eluting in a solvent) from a 1R6F reference cigarette. In this study, the neutral red uptake (NRU) assay (human bronchial epithelial (BEAS‑2B) and human liver (HepG2) cells), the Ames test (in five bacterial strains, +/‑S9 metabolic activation), and the *in vitro* micronucleus (IVM) formation assay (Chinese hamster lung fibroblast (V79) cells, +/‑S9), were performed to respectively assess the cytotoxicity, mutagenicity, and genotoxicity, of the tested products’ extracts. Cytotoxicity evaluations in the NRU assay were based on EC_50_ (the concentration effective in producing 50% of the maximal cytotoxic response), or if not achieved, an EC_20_ (20% of the maximal cytotoxic response). Statistically significant cytotoxicity was observed for all extracts, as determined by achievement of >20% cytotoxicity in both cell lines in the tested concentration range. However, extracts from the two tested TFNPs and snus (only in BEAS‑2B cells) did not reach the EC_50_ at the top testing concentration (10 mg/mL). Cytotoxicity was orders of magnitude (167- to 798‑fold) lower for the snus and TFNP extracts compared with that of 1R6F TPM. Additionally, in this study, TFNP and snus extracts were not mutagenic nor genotoxic compared with 1R6F TPM extracts. Overall, these data substantiate evidence supporting a reduced toxicological impact of TFNPs compared to cigarettes.

Shaikh et al. [39] assessed the potential toxicity of ONDS extracts on human gingival epithelial progenitor (HGEPp) cells and 16‑HBE and BEAS‑2B cells from lung bronchial epithelium, focussing on the impact of different flavours on toxicity. Gingival HGEPp cells were treated for 24 hours with extracts from two brands of flavoured snus ("General Snus" (classic original), "Nick & Johnny" (Americana), and four brands of TFNPs (ZYN (smooth tobacco, 6 mg), Grizzly (wintergreen menthol, N/A mg; noting that this product is actually a moist snuff product and not a TFNP), "lucy" (spearmint, 8 mg), and "on!" (citrus fruit, 8 mg)). Lung bronchial epithelial 16‑HBE and BEAS‑2B cells were treated with extracts from a single spearmint snus with an unspecified nicotine content and four TFNPs ("on!" (original flavour, 8 mg), "Rogue" (mango, 6 mg), "Velo" (black cherry, 7 mg), and "ZYN" (cool spearmint, 6 mg)). The authors then measured inflammatory cytokine (TNF‑$\alpha$, IL‑6, and IL‑8) release, cellular reactive oxygen species (ROS) production, and cytotoxicity. Shaikh et al. reported that flavoured ONDS extracts elicited differential toxicities in a dose‑ and flavour-dependent manner, with fruit extracts resulting in the highest cytotoxicity [39]. Tobacco‑ and fruit‑flavoured, but not menthol‑flavoured, ONDS exhibited increased ROS production, and flavoured ONDS led to differential cytokine release, which also varied by flavour (menthol, tobacco, or fruit) and nicotine strength. Overall, while between‑flavour differences were observed, these differences were only assessed relative to negative control, and the statistical significance of the differences between flavours was not evaluated. Moreover, the study did not assess the effects in the assays relative to cigarette smoke, as was performed in other studies [34,38,40], misclassified at least one of the study products, and did not include any pouches containing synthetic nicotine. These methodological issues may prevent firm conclusions from being drawn from the study data.

In the studies described above, TFNP extracts were generated using tissue culture media or saline. In a slightly different approach, Miller‑Holt et al. [40] investigated the *in vitro* toxicological potential of extracts from three variants of "Nordic Spirit" TFNPs, which were generated using complete artificial saliva (CAS). These extracts were compared with CAS extracts from the CORESTA CRP1.1 reference smokeless tobacco (snus) product and smoke condensed from the 1R6F reference cigarette. In NRU, Ames, and IVM assays, all TFNPs and the reference snus product extracts were observed to be less cytotoxic, less mutagenic, and less genotoxic, compared with those of 1R6F reference cigarette smoke extracts. In addition, toxicity endpoints were similar between the snus and TFNP extracts. Data from studies using CAS extracts from the products assessed also failed to be classified as irritants (as measured using the MTT assay) in an EpiGingival™ 3D tissue model [40].

The assessment of chemical constituents and toxicological properties of TFNPs should be conducted using robust and reproducible *in vitro* dissolution methods. By employing these methods, estimations of the release profile of nicotine and other constituents can be determined under controlled laboratory conditions, closely mimicking the actual dissolution process that occurs in the mouth. The method provides accurate data on the rate and extent of nicotine release, offering valuable insights into the product's behaviour. Using a method developed and validated of testing dissolution for nicotine release from smokeless tobacco products [41], Aldeek et al. [42] assessed commercially available "on!" and "ZYN" TFNPs across seven flavours and five nicotine strengths and traditional smokeless tobacco products. The cumulative release of nicotine and percent of total dissolution across all on! TFNPs increased proportionally to the pouch nicotine content. Additionally, faster nicotine dissolution (>80%) was observed between 0 and 20 minutes, and >95% release was achieved within 40 minutes before reaching a plateau. Reported values of difference (f1) and similarity (f2) factors with different nicotine strengths and flavours suggested equivalency between all the tested on! pouches and that the pouch nicotine content did not impact the nicotine release profile. When comparing an on! Wintergreen 3.5 mg nicotine TFNP with the traditional smokeless tobacco products, equivalency was seen between the percentage of nicotine released at each collection time point, and the rate of nicotine release from Skoal smokeless tobacco pouches was slower than that observed for the on! TFNP. When comparing nicotine strengths and flavours, similar amounts of nicotine release were reported although the nicotine release rate for the on! 3.5 mg nicotine TFNP was slightly slower than observed for the ZYN 3 mg nicotine TFNP. Overall, these data suggest that nicotine release from TFNPs may be determined by characteristics intrinsic to each particular TFNP.

In summary, data from the chemistry and in vitro toxicological assessments described above suggest that TFNPs may present reduced exposure to harmful chemicals compared to cigarette smoke and have greater THR potential than smokeless tobacco products (such as snus), recognised to offer reduced relative risk compared with cigarette smoking [43-45]. Some authors [28,32] also suggest that the exposure profile of TFNP use may approximate that of the NRT use. Additionally, most studies have shown that TFNP flavours have no measurable impact on toxicological endpoints. However, one study did appear to report a flavour impact [39]. Although methodological constraints may preclude drawing too much insight from those findings, they do perhaps indicate a need for further assessment of the potential effect of flavours and the examination of different commercially available products, since there may be differing effects between flavour types and ingredients. In this regard, it is notable that intrinsic product characteristics may influence the release of chemicals into TFNP extracts. Further chemical and toxicological characterisation studies are required to assess the impact of different TFNP products with respect to constituents, flavours, and nicotine content. In addition, and to make data more comparable across studies and between products, the establishment of standardised assessment approaches for the toxicological characterisation of TFNPs, including standardised approaches to the generation of TFNP extracts, and using comparable limits of quantification across studies, would be beneficial.

Nicotine Pharmacokinetic Studies Using TFNPs

THR is not solely achieved by creating a product that is reduced risk compared to a cigarette. The product also needs to be accepted and used by adult smokers to help them transition away from smoking. One of the key questions related to TFNPs is whether they can provide satisfactory nicotine delivery and generate positive subjective effects in adult smokers seeking an alternative to cigarettes. These factors can be integrated to form an assessment of the acceptability of a tobacco/nicotine product for adult smokers and can determine whether a product has THR potential while assessing the potential abuse liability of a product [46,47]. To address this question, several groups have investigated nicotine pharmacokinetics (assessment of the blood nicotine profile during and after use of tobacco/nicotine products; six studies), the relative bioavailability of nicotine (the amount of nicotine that enters the body during TFNP use relative to that seen during lozenge and nicotine gum use; one study), and subjective effects of TFNPs (seven studies). When considering the nicotine pharmacokinetic studies, it is important to note that C_max_ (the maximum level of nicotine in the blood observed following product use) and T_max_ (the time to reach the maximum blood nicotine concentration) should ideally not be higher and lower than those for cigarettes, respectively, since this could potentially indicate an equal or higher abuse liability that would not support THR [46,47].

Lunell et al. [48] evaluated ZYN TFNPs, compared with Swedish snus and American moist snuff, in two studies assessing nicotine pharmacokinetics among healthy users of tobacco‑containing snus. The first study compared nicotine pharmacokinetics following a 60‑minute use of a single ZYN ("Smooth", unflavoured) TFNP containing either 3 or 6 mg nicotine with that following a 60‑minute use of a single "General Snus" 8 mg pouch (flavours not reported). The second study compared nicotine pharmacokinetics following the use of a single ZYN 8 mg TFNP compared with 16 mg (two 8 mg pouches were placed in the mouth simultaneously), General Snus pouches, and 18 mg "American Longhorn" moist snuff. In the first study, the C_max_ for ZYN 3 mg was significantly (25%) lower than that for 8 mg General Snus, whereas C_max_ for ZYN 6 mg was significantly (42%) higher. In addition, the area under the plasma nicotine concentration‑time curve (AUC, a measurement that estimates total nicotine exposure) for ZYN 3 mg was 27% lower than that for General Snus 8 mg. However, the AUC for ZYN 6 mg was significantly (34%) greater than for 8 mg General Snus. The *in vivo* extracted amount of nicotine (the amount of nicotine remaining in the pouch after use subtracted from the amount in the unused product) was statistically different between each pouch: 1.5 mg extracted per pouch for ZYN 3 mg, 2.4 mg per pouch for 8 mg General Snus, and 3.5 mg per pouch for ZYN 6 mg. The extracted fractions of nicotine for both 3 and 6 mg ZYN products (56% and 59%, respectively) were significantly higher compared with General Snus 8 mg (32%). In the second study comparing a ZYN 8 mg TFNP with snus and moist snuff, similar plasma nicotine concentration curves, AUC, and C_max_, were observed for ZYN 8 mg and Longhorn Natural 18 mg moist snuff. ZYN 8 mg TFNP use elicited 17% lower AUC and 12% lower C_max_ values than the use of two General Snus 8 mg pouches. The amount of nicotine extracted from two General Snus 8 mg pouches was significantly higher than that from the other products; however, the extracted amount of nicotine from the ZYN 8 mg TFNP (3.8 mg extracted per pouch) was significantly higher than that from Longhorn Natural 18 mg moist snuff (3 mg extracted per pouch) but lower than the extracted amount of nicotine from General Snus 2 × 8 mg pouches (2.5 mg extracted per pouch). The extracted fraction of nicotine for the ZYN 8 mg TFNP (50%) was higher than for Longhorn Natural (19%) and two General Snus 8 mg pouches (33%) (Table 2). These data suggest that the two higher doses of ZYN (6 and 8 mg) deliver nicotine as quickly and to a similar concentration compared with existing smokeless products (Table 2), with no significant adverse effects.

**Table 2:** Example Data for Nicotine Product Pharmacokinetic Parameters. Summary of mean nicotine pharmacokinetic parameters for oral nicotine products from Lunell et al. [48] compared with publicly available data for a conventional cigarette [49]. AUC, the area under the plasma nicotine concentration‑time curve; C_max_, the maximum level of nicotine in blood plasma seen following product use; T_max_, the time to reach the maximum plasma nicotine concentration. Note that the data reported by Lunell et al. [48] are not baseline subtracted compared with those reported by Chapman et al. [49], although, in the former paper, baseline plasma nicotine levels were low and baseline subtraction had little impact on the study findings [48].

Product	AUC (ng/mL*h)	C_max_ (ng/mL)	T_max_ (minutes)
Conventional cigarette	23.16	17.81	7
ZYN 3 mg	32	7.7	61
ZYN 6 mg	57.7	14.7	66
ZYN 8 mg	58.4	18.5	59
Nicorette gum 4 mg	52.5	12.8	46
General Snus 8 mg	45.9	10.6	69
General Snus 2 x 8 mg	70.3	21.2	63
Longhorn Natural 18 mg	60.6	16.9	65

Rensch et al. [50] investigated the nicotine pharmacokinetics of on! TFNPs (4 mg nicotine per pouch with six flavour variants) compared with each subject’s own brand cigarettes (OBC). Additionally, participants were asked to self‑report the subjective effects of tobacco/nicotine withdrawal, direct effects of using the product, smoking urges, and intent to use the product again. The pharmacokinetic assessments comprised a single product use session in which one TFNP was used for 30 minutes, or one cigarette was smoked in 5 minutes. C_max_ values for all six on! TFNPs were significantly lower (range: 9.1-11.5 ng/mL) compared with that for OBC (16.3 ng/mL), while T_max_ was significantly longer (30.1-34.9 minutes) than OBC (7.5 minutes). The AUC was similar between products, ranging from 860 to 1,118 ng·min/mL for the TFNPs compared with 1,008 ng·min/mL for OBC. Positive subjective effects (e.g., pleasant, satisfying, calm, awake) were lower for TFNPs compared to OBC. The ability of a product to reduce the urge to smoke was lower for TFNPs than for the OBC, and the time course of urge reductions occurred later for TFNPs than for the cigarettes. For both nicotine pharmacokinetics and subjective effects, there were no differences observed between flavours. In agreement with previous studies [46,47], data showing the delivery of nicotine and the elicitation of positive subjective effects suggest that on! TFNPs may have some ability to facilitate switching from smoking to using TFNPs while having lower abuse liability/addiction potential than cigarettes.

McEwan et al. [51] assessed nicotine pharmacokinetics and subjective effects of five commercially available TFNPs (Lyft (mint, 10 mg), ZYN (spearmint, 10 mg), Nordic Spirit (mint, 9 mg), Skruf Super White (mint, 8 mg), on! (mint, 6 mg)) and a commercially available cigarette ("Pall Mall Red"). In this study, healthy adults who were current dual users of snus (for ≥6 months) and cigarettes (≥5 per week) used one study product each day for a defined period following overnight nicotine abstinence. In line with previous studies, T_max_ was reached shortly after the end of the five‑minute smoking session for the cigarette and after the end of the 60‑minute session for the TFNP. The authors reported that the C_max_ for the TFNPs ranged from 11.9 to 18.4 ng/mL, compared with 13.9 ng/mL for the cigarette, and the AUC ranged from 35.8 to 53.7 ng·h/mL for the TFNPs compared with 25.2 ng·h/mL for the cigarette. Regarding the subjective effects measures, scored from 0 (strongly dislike/not at all) to 10 (strongly like/very much), scores for product liking and intent to use the product again were the highest for the cigarette, though it is important to note that large proportions of subjects reported product liking and intent to use again scores greater than 5, and the likelihood of such scores was generally increased as pouch nicotine content increased, although product liking was not directly related to nicotine delivery based on the pharmacokinetic findings of the study. on! TFNPs reportedly exhibited significantly lower scores for product liking and the intent to use compared to the cigarette, but there were no further significant differences between the products in the subjective effects scores. Similar to the findings of Rensch et al. [50], this study suggests that TFNPs may provide an acceptable and viable alternative to cigarettes while providing, at least for some products, lower abuse liability due to lower C_max _and higher T_max_ values. Furthermore, intrinsic physical differences between different TFNP, including nicotine content, may influence this ability to act as a smoking alternative.

**Figure 3:**
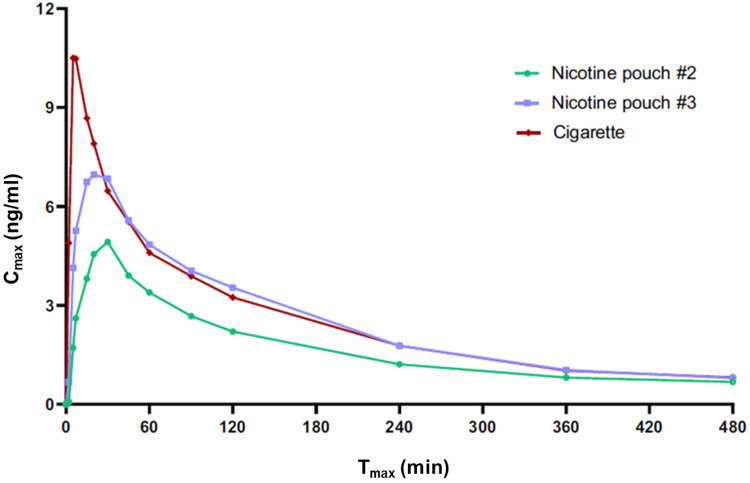
Example of Nicotine Pharmacokinetic Data for TFNPs and Cigarettes. Baseline adjusted nicotine levels measured in the plasma of adult traditional tobacco product users during 8 hours following the use of a single TFNP (ZoneX #2 or ZoneX #3) or a cigarette. Copyright/license: This figure has been adapted from Chapman et al. [49], which is an open-source article distributed under the terms and conditions of the CC BY license.

In a similar study by Chapman et al. [49], pharmacokinetic, pharmacodynamic, safety, and tolerability profiles of two "ZoneX" TFNP variants (ZoneX #2 and ZoneX #3; 5.8 mg and 10.1 mg nicotine per pouch, respectively) were compared with those of a commercially available cigarette in dual users of cigarettes and Scandinavian snus. A single TFNP was used for 20 minutes (according to the manufacturer’s instructions), and a single cigarette was smoked for approximately five minutes. A significantly higher C_max_ (11.6 ng/mL) and shorter T_max_ (8.5 minutes) were reported for the cigarette compared with the ZoneX #2 and ZoneX #3 TFNPs (C_max_ 5.2 and 7.9 ng/ml; T_max_ 26 and 22 minutes; Figure 3). The AUC was significantly lower for the ZoneX #2 TFNP compared with the other two study products, which were not significantly different from one another. All products effectively reduced subjects’ urge to smoke and presented favourable product liking scores. The authors also reported that TFNPs were well tolerated following short‑term use with no serious adverse events reported. This study provides further evidence of the potential ability of TFNPs to act as an acceptable alternative for cigarettes while possessing lower abuse liability/addiction potential.

Liu et al. [52] assessed nicotine pharmacokinetics and subjective effects of five mint‑flavoured on! TFNPs with various nicotine contents (1.5 mg, 2 mg, 3.5 mg, 4 mg, and 8 mg nicotine per pouch) relative to subjects’ OBC and own brand moist smokeless tobacco (OBMST) in healthy adult dual users of cigarettes and moist smokeless tobacco. For OBCs, a single was smoked by taking 10 inhalations at approximately 30‑second intervals, and the use regimen for the TFNP and OBMST involved using either a single TFNP or a single portion (approximately 2 g) of OBMST for 30 minutes. The T_max_ for all TFNPs was higher (range: 32.5-33.9 minutes; that is, the time to peak was longer) than OBC (8.5 minutes) and similar to OBMST (34.4 minutes). The C_max_ and AUC values for the TFNPs increased with increasing nicotine content. Thus, the AUC for the 8 mg on! TFNP (1,441 ng·min/L) was significantly higher than that for cigarettes (803.1 ng·min/L) and smokeless tobacco (987.7 ng·min/L). Furthermore, for the 8 mg on! TFNP, the C_max_ (14.5 ng/mL) was significantly higher than that for cigarettes (10.5 ng/mL) and smokeless tobacco (9.2 ng/mL). Reductions in the urge to smoke were significantly higher for cigarettes than for the 1.5 and 4 mg on! TFNPs and not significantly different between the cigarette and the other (2, 3.5, and 8 mg) on! TFNPs. Reductions in cigarette craving for the 1.5, 2, 3.5, and 4 mg on! TFNPs were significantly lower than that of cigarettes, but not the 8mg on! TFNP. Compared with smokeless tobacco, reductions in cigarette craving were significantly lower for the 1.5, 2, 3.5, and 4 mg on! TFNPs, and reductions in the urge to smoke were significantly lower for the 1.5 and 4 mg on! TFNPs. Consistent with the other studies described above, these data demonstrate the ability of TFNPs to effectively deliver nicotine and to potentially provide a viable alternative to cigarette smoking, with a lower abuse liability due to the lower C_max_ and higher T_max_, though this may not necessarily be the case for some products with high nicotine content for which C_max_ can be higher than that for cigarettes.

Azzopardi et al. [53] compared nicotine pharmacokinetics and product satisfaction between a prototype TFNP with 4 mg nicotine per pouch with NRT gum and lozenge products (both 4 mg nicotine) in adult smokers. In this study, subjects placed a single TFNP in their mouth for 60 minutes, placed a single piece of nicotine gum in their mouth, and chewed and "parked" it once a minute for 30 minutes, or placed a lozenge in their mouth and moved it from side to side for approximately 10 minutes until it had completely dissolved. The C_max_ for the TFNP, the nicotine lozenge, and nicotine gum were 8.5, 8.3, and 4.4 ng/mL, respectively, and the AUC values were 30.6, 31.5, and 14.3 ng·h/mL, respectively. The T_max_ was similar across the study products (median range: 0.83-1.00 hour). Based on these values, the authors suggested that the TFNP showed similar nicotine bioavailability to the nicotine lozenge and superior bioavailability to the nicotine gum. Compared with the nicotine lozenge, the TFNP appeared more satisfying, with a higher number of positive responses to subjective satisfaction questions.

Thornley et al. [54] measured the relief from tobacco withdrawal symptoms and satisfaction among smokers using the "Zonnic" TFNP compared with NRT gum with a comparable nicotine content and a placebo (zero nicotine) pouch. The authors reported stronger craving reductions for the TFNP compared with both NRT gum and the zero nicotine TFNP. The TFNP was also more effective than NRT gum in reducing irritability, though no differences were observed for other subjective effects such as restlessness and difficulty concentrating. In addition, abstinence from cigarette smoking over an approximately 10‑hour ambulatory study period was higher following TFNP use compared with NRT gum or the placebo TFNP. Specifically, the odds ratio for abstinence was almost three times greater for the TFNP than the NRT gum, potentially due to superior subjective effects domains such as "helpful in abstaining from cigarettes", "pleasant to use", and "satisfaction". These findings are important since they allude to a beneficial effect, through subjective effects, of TFNPs in aiding smoking abstention, and perhaps complete switching away from cigarettes, which is greater than that of NRT gum. However, this potential, in terms of long‑term abstention, was not found in a clinical trial that offered TFNPs to smokers as part of a "selection box" of various nicotine‑containing products [55], in which very few subjects (<4%) chose to use the TFNP. It is notable that the studies by Thornley et al. [54] and Walker et al. [55] were conducted some years ago. Advances in science and technology have enabled innovation and the creation of TFNP products with better nicotine delivery and subjective effects profiles. This perhaps highlights the need for more studies examining the potential for modern TFNPs to support smokers transition away from cigarettes.

In their totality, data from nicotine pharmacokinetic assessments suggested that TFNP use typically increases blood nicotine levels to a lesser degree than cigarette smoking, although this is dependent on the physicochemical characteristics of each product, including its nicotine content, and that this nicotine delivery occurs more slowly for TFNP. While the nicotine content appears to be a prime determinant of nicotine delivery, other factors such as pouch composition likely also play a role. In addition, TFNP nicotine content appears to have little relation to nicotine delivery when comparing different brands with different pouch compositions. Furthermore, in all studies, the C_max_ occurred in conjunction with pouch removal, showing that usage time is a major factor affecting nicotine delivery. It is also apparent, again with differences between individual pouches, that TFNPs can elicit reductions in craving, urges to smoke, withdrawal symptoms, and other positive subjective effects such as satisfaction and relief, among smokers. While these effects are generally smaller than those observed with cigarette smoking, and, as for nicotine delivery, this may be dependent on product characteristics, they do add to the weight of evidence that TFNPs may provide a viable and acceptable alternative to cigarettes for adult smokers and facilitate their switching to potentially less harmful nicotine products. While data from switching studies are encouraging, further studies are required for a greater understanding of the ability of TFNPs to support complete switching away from smoking, including examining the ability of TFNPs with different characteristics (e.g., flavour, nicotine content) to support smoking abstinence.

Studies Assessing Biomarkers and Disease/Health Endpoints Related to TFNP Use

Several studies have assessed the potential impact of TFNP use in humans, in some instances using biomarkers of exposure (BoE, indicators of exposure to harmful toxicants, one study) and biomarkers of potential harm (BoPH, indicators of disease risk, three studies), which are indicative of, or related to, potential health effects [56]. Other studies have either assessed disease/human health endpoints, such as damage to the oral mucosa or autonomic nervous system (ANS) dysfunction, or modelled the impact of a TFNP on all-cause mortality relative to smoking. Findings from such studies may be indicative of the likelihood that TFNPs can contribute to THR.

Using a cross‑sectional study design, Azzopardi et al. [57] assessed BoE and BoPH among exclusive users of "Velo" TFNPs compared with current smokers, former smokers, and never smokers. In the study, the levels of eight biomarkers of exposure to certain priority toxicants identified by the World Health Organization TobReg group [30] and seven biomarkers of potential harm related to lung disease, cardiovascular disease, and inflammation/oxidative stress were examined. In addition, subjects’ self‑reported oral health and quality of life were assessed by completing brief surveys. Seven of the eight biomarkers of exposure were significantly lower in the TFNP user group compared with the current cigarette smokers group, by between 23% and 97%, and total nicotine equivalents (a marker for overall nicotine delivery) were significantly higher among TFNP users compared with cigarette smokers. Among these biomarkers, the biomarker NNAL, which is indicative of exposure to the lung carcinogen NNK and is also considered a biomarker of potential harm, was 91% lower among Velo TFNP users compared with current smokers. Data suggested that the residual 9% NNAL among those study participants who switched to using TFNPs stems from the long (up to 18 days [58]) terminal half-life of NNAL, such that the residual NNAL detected is a legacy of smoking before switching to TFNP use or short periods of relapse during the study period. Potentially also, although the study recruited exclusive TFNP users, there is the potential that residual NNAL could stem from snus use not reported by study subjects. Biomarkers of potential harm measured in this study were reportedly lower or comparable between Velo TFNP users and cigarette smokers. COHb (an indicator of carbon monoxide exposure linked to the development of cardiovascular disease), white blood cell count (linked to inflammation), sICAM‑1 (an inflammation marker linked to cardiovascular disease), and 11‑dTX B2 (a cardiovascular disease marker) were 46%, 19%, 9%, and 18%, respectively, lower in the TFNP group compared with the current smoker group. 8‑epi‑PGF2$\alpha$ (a biomarker of oxidative damage) and HDL‑C (a blood lipid marker linked to cardiovascular disease risk) were lower in the Velo TFNP user group compared with the current smoker group, though authors note that these were highly variable and not statistically significant. Fractional exhaled nitric oxide (FeNO, a marker of lung damage that is lower among smokers) was 97% greater in the TFNP user group compared with the current smoker group. Furthermore, self‑reported oral health and quality of life were comparable or improved in Velo TFNP users compared with cigarette smokers. While this study had a limitation in that the average age of subjects in the Velo TFNP user group was much lower than that of the current smokers, these data overall suggest a lower health impact of TFNP use and favourable changes in biomarkers among smokers who switch completely to using TFNP and, therefore, show beneficial effects of switching and the potential for THR.

Alizadehgharib et al. [59] investigated the potential effects on the oral mucosa when regular Swedish snus users substituted their snus use with ZYN TFNPs during a six‑week observational period. After examining for the presence and severity of oral mucosal lesions at the site of the pouch at the baseline visit and, subsequently, at the follow‑up visits after two, four, and six weeks, a statistically significant decrease in the severity of lesions was observed between visits compared with baseline and the authors stated that all of the TFNPs were considered safe and well tolerated. The improvement in oral lesion severity correlated with the degree of substitution of snus with TFNP. Additionally, this study compared TFNP, and snus extracts *in vitro* by measuring the production of pro-inflammatory cytokines from peripheral blood mononuclear cells (PBMCs). PBMCs were isolated from 11 healthy blood donors and exposed for 24 hours to supernatants obtained by diluting 1 g of the contents of four snus variants and seven TFNP variants (six ZYN TFNPs and a single Lyft TFNP used as an additional control) in tissue culture medium. PBMCs were then exposed to these extracts at a concentration of 0.025 g/mL, and the production of 48 inflammatory cytokines was assessed. All four snus products were assessed, and the Lyft TFNP demonstrated a significant increase in the production of pro-inflammatory cytokines IL‑1$\beta$, IL‑6, IL‑18, TNF‑$\alpha$, and MIP‑1$\alpha$, compared with unstimulated cells, and these effects on cytokine production were lower for the ZYN TFNPs than for the snus products. Overall, data from the clinical and *in vitro* studies in this publication suggest reduced impacts of TFNPs on oral lesions and on the release of pro-inflammatory cytokines compared with tobacco‑containing snus.

The activity of the ANS, a component of the peripheral nervous system that regulates involuntary physiologic processes, including heart rate, blood pressure, respiration, and digestion, was examined by Menshov et al. [60]. The authors assessed the heart rate variability (HRV) among smokers, non‑smokers, and former smokers when they used various types of tobacco and nicotine products, including a TFNP (Lyft), cigarettes, an EVP, and a HTP. TFNP use was associated with a prolonged (40-minute) increase in heart rate, which, according to the authors, is “understandable”, considering the longer period of use in the oral cavity compared to other products tested. However, the overall impact on HRV was the lowest of all the nicotine product tests when subjects used TFNPs. It is unclear if, in this study, the authors standardised product nicotine content and usage profiles, which have the potential to influence outcomes.

Cigarette smoking reportedly causes staining to teeth [61,62]. Dalrymple et al. [63] examined how Lyft TFNP extracts compared to extracts from a 1R6F reference cigarette, a HTP, EVP aerosol, extracts from a CORESTA CRP1.1 reference snus product, coffee, and wine using a bovine *in vitro *model to measure tooth staining over a three‑month exposure period. Cigarette smoke particulate, snus extract, coffee, and wine induced statistically higher levels of staining than TFNP extracts, and staining levels following TFNP extract exposure were similar to the negative control. While this study suggests that switching to the use of TFNPs could have cosmetic benefits for smokers, this study did not assess reductions in staining over time subsequent to smoke staining. Therefore, given the present data, the benefit may be restricted to preventing the worsening of staining and not improving teeth colour following switching. 

Although they did not directly assess the health impacts of TFNP use, Lee et al. [64] used a dynamic population microsimulation model to estimate the effect that the hypothetical introduction of the ZYN TFNP may have on the combined distribution of smoking and ZYN use and on overall mortality, among the US population from the year 2000 (baseline) to the year 2050. The model included two scenarios: a "base case" where ZYN was never introduced and a "modified case" where ZYN was introduced immediately after baseline. In both scenarios, informed assumptions concerning initiation, quitting, and switching rates were made. The model estimated that the prevalence of current cigarette smoking fell steadily from about 22% at baseline to approximately 10% for the base case and to approximately 7% for the modified case. Additionally, in the modified case, the prevalence of ZYN use (alone or with cigarettes) increased from zero at baseline to 2.5% after 50 years. The model also estimated that the reduction in total and product‑related deaths when scaled up to the whole US population at ages 35-84 was approximately 320,000 and 700,000, respectively. In a sensitivity analysis, assuming that the reduction in excess mortality due to ZYN use is 20% from smoking, compared with the 3.5% excess mortality risk assumed in the main analyses, the reduction in product‑related deaths was still approximately 600,000. These data are suggestive of a marked population health benefit of the market introduction of a TFNP.

Overall, studies assessing the potential health impacts of TFNP use have demonstrated reduced exposure to harmful chemical toxicants compared with exposure due to cigarette smoking, favourable changes in indicators of disease risk, and significant reductions in oral mucosal lesions (Table 3). Interestingly, data from both *in vitro* studies and human clinical assessments suggest that there is a reduced inflammatory response to TFNP extracts compared with other, tobacco‑containing products. Overall, these data are suggestive of a positive individual health benefit of exclusively switching from both combusted and non‑combusted tobacco products to TFNPs. At the population level, the modelling study of Lee et al. [64] suggests a large population health benefit, in terms of reduced mortality, of the introduction of TFNPs into a marketplace. This is perhaps to be expected, given the switching potential, the reduced toxicant exposure profile, reduced toxicological impact reported *in vitro*, and the low appeal to nicotine non‑users, described earlier in this review. Overall, the number of studies conducted to date is small, and further examination of the potential impacts of TFNPs on various health endpoints and at the population level is warranted. The studies reviewed here add to the weight of evidence of a lower relative risk of TFNP use compared with cigarette smoking. Therefore, TFNPs may be a reduced-risk alternative for smokers who completely switch from smoking.

**Table 3:** Example Data From Studies Suggesting Changes in Health Indices Among Tobacco Product Users Switching to Using TFNPs. Increased (↑), reduced (↓), or no significant change (=) in biomarkers of exposure (BoE) and biomarkers of potential harm (BoPH) associated with TFNP use compared with cigarettes, HTPs, EVPs, snus, or controls. Abbreviations: CC, combustible cigarette; HTP, heated tobacco product; EVP, electronic vapour product

Reference	Biomarker	Study design	Comparison	Effect
Azzopardi et al. [28]	Selected BoE	Clinical	CC	↓
	Selected BoPH	Clinical	CC	↓ / =
Alizadehgharib et al. [59]	Oral lesions	Clinical	Snus	↓
	Pro-inflammatory cytokines	In vitro	Snus	↓
	Pro-inflammatory cytokines	In vitro	Control	↑/↓
Menshov et al. [60]	HRV	Clinical	CC, HTP, EVP	↓
	HRV	Clinical	Control	↑
Dalrymple et al. [63]	Tooth staining	In vitro	CC, HTP, EVP, snus	↓
	Tooth staining	In vitro	Coffee, wine	↓
	Tooth staining	In vitro	Control	=

TFNP Sales Data

Assessing TFNP sales data is important in understanding the prevalence and frequency of TFNP use. Two studies were identified that assessed TFNP sales, both of which analysed data collected in the US. Majmundar et al. [65] assessed Nielsen TFNP sales data and trends by volume and nicotine levels in the US market between August 2019 and March 2022. In total, this analysis examined data from 2,182 local trade areas across 48 US states and Washington DC. Four product brands were identified in the sales data and market research: ZYN, Rogue, on!, and Velo. All sales were aggregated by month and year, with the authors defining one unit as one pouch due to differences in pack size. Overall, TFNP sales increased from 126 million units in August to December 2019, to 808 million units from January to March 2022. ZYN led the overall market share with 58.8%, followed by on! with 24.6%, Velo with 12.1%, and Rogue with 4.8%. Regarding pouch nicotine content, TFNPs with 6 mg (1,365 million units), 4 mg (470 million units), and 3 mg (450 million units) nicotine per pouch were most commonly sold during the study period. However, sales of products with 8 mg of nicotine per pouch increased more rapidly than products with lower nicotine levels. Overall, the most recent data from 2022 suggest that the most commonly sold TFNP contained 6 mg of nicotine.

Rose et al. [66] evaluated the neighbourhood distribution of availability of newer tobacco products, including TFNPs, across four states (New Jersey, Kentucky, North Carolina, and New York) in the US in 2021. Standardised store audits were conducted across 242 tobacco retailers, with each retailer geocoded with census tract demographics. Regression analysis was then performed to assess the availability of each product with correlates of the proportion of non‑Hispanic white residents, households in poverty, proximity to schools, site, and store type. On average across the four states, half of the stores audited sold TFNPs (76% in Kentucky, 46% in North Carolina, 43% in New York, and 37% in New Jersey). TFNPs were less commonly available in all store types relative to chain convenience stores, in deprived areas, in areas with a low proportion of non-Hispanic white residents, and in stores near schools. Additionally, the availability of nicotine pouches was higher in Kentucky compared with New Jersey. The authors concluded that the availability of TFNPs is more likely in neighbourhoods with a greater percentage of non‑Hispanic White residents as a means of targeting sales towards those who already use smokeless tobacco and may want to switch away from using these products to using a potentially reduced harm alternative.

Use Prevalence and Intentions to Use TFNPs

To support THR strategies, potentially reduced-risk products need to be both accepted and satisfying to adult smokers and support their transition away from cigarette smoking while having minimal appeal or use among unintended users. Therefore, understanding the appeal of TFNPs among those who are nicotine‑naïve, including both adults and youth, compared with the appeal of these products for adult smokers seeking an alternative to cigarettes, is paramount to assessing their public health potential [20]. Robichaud et al. [19] emphasised the necessity of monitoring product use, and the marketing of TFNPs in order to ensure nicotine use among non‑smokers, especially youth, does not occur. This is particularly necessary since some studies have reported that the variety of fruit flavours, and the ability of TFNPs to be used discreetly, could appeal to non‑users of nicotine, including youth [67]. The studies reviewed in this section focus on intentions and motivation to use TFNPs across the general population, including both youth and adults. Additionally, the relative and absolute risk perceptions of TFNPs are examined since these may be a driver of product uptake, since intentions to use and actual use of any given tobacco/nicotine product are negatively impacted by increased perceptions of the risks associated with their use.

Motivation and Intention to Use TFNPs Among Youth

Harlow et al. [68] analysed data from an ongoing survey study of behavioural health among 3,516 Southern Californian adolescents in the ninth and 10th grades [69] to assess the prevalence of ever use and past six-month use of TFNP, other non‑tobacco oral nicotine products (i.e., gum, lozenges, tablets, and/or gummies), EVPs, cigarettes, hookah or waterpipe, cigars, cigarillos, and snus between September and December 2021. Among the sample, the prevalence of use was the highest for EVPs (ever use was 9.6%; past six‑month use was 5.5%), followed by non‑tobacco oral nicotine products (ever use of 3.4%; past six‑month use of 1.7%), and <1% for other products, including past six‑month TFNP use. Although the authors suggest that TFNPs were the second most prevalent nicotine product used by adolescents (behind EVPs), this definition included all oral product use and not just TFNP use specifically.

Using the same dataset from the Southern Californian adolescent survey study collected from September to December 2021, Tackett et al. [70] examined whether the willingness of adolescents to use ONDS differed by product type and flavour and whether sociodemographic disparities existed in the use of these products. Among 1,289 demographically diverse adolescent never-tobacco product users in the ninth and 10th grades, participants were shown random images of either fruit or mint smokeless tobacco (snus), non‑medicinal nicotine gums and lozenges, TFNPs, and nicotine gummy products and were questioned on their willingness to try if offered to them by a friend or someone they trust. Although absolute willingness to try values were not reported, willingness to try TFNPs appeared to be lower than that for the other non‑tobacco nicotine products assessed, though it was significantly higher than that for smokeless tobacco (snus). In addition, while the effect size was modest, authors reported that mint flavours appeared preferable over fruit flavours across all study products.

In a third analysis of the dataset of a Southern Californian adolescent survey study (September to December 2021), Vogel et al. [71] compared susceptibility to smoke cigarettes, use EVPs, or use novel flavoured ONDS (nicotine gum, lozenges, and gummies) marketed as “tobacco‑free”, among 3,129 never‑users of nicotine products. Susceptibility was assessed using three self‑report items: willingness to try the product if offered by a best friend, intention to use the product in the next year, and curiosity about the product. The authors reported that most participants were not susceptible to the use of inhalable or oral nicotine products (73%), with only 13% susceptible to both types of products, 11% susceptible to inhalable nicotine products only, and 3% susceptible to oral nicotine products only. Considering specific products, susceptibility was the highest for EVPs (19.7%), followed by cigarettes (15.0%) and nicotine gum, lozenges, tablets, and/or gummies (15.0%), and the lowest for TFNPs (8.7%).

In cross‑sectional analyses of data from a separate Californian youth tobacco survey study dataset (November to December 2021), Han et al. [72] reported on beliefs about using ONDS and interest in using TFNPs and smoking cessation medications to reduce/quit vaping. Analyses were conducted among a sub‑cohort of 1,460 current EVP users with motivation to quit EVP use and who did not use ONDS, as well as in those without motivation to quit EVP use and EVP non‑users. Past 30‑day ONDS use was low overall, but significantly more common among past 30‑day EVP users with (4.6%) or without (5.7%) quit motivation compared with EVP nonusers (0.4%). Additionally, the prevalence of ONDS use and the total number of positive beliefs were higher in the past 30‑day EVP users (either with or without quit motivation). EVP users with or without quit vaping motivation were more likely than non-users to perceive that ONDS are more affordable than other nicotine products, less harmful than smoking/vaping, available in appealing flavours, convenient to use, and able to be used in places where smoking/vaping is not allowed. The prevalence of believing that ONDS can help people quit smoking/vaping was reported to be higher among EVP users with quit motivation (18.4%) than among non‑users (9.1%), but not significantly different from EVP users without quit motivation (15.9%). Interest in using ONDS to reduce/quit vaping was higher among those with low/moderate (vs. high) self‑efficacy in quitting vaping (adjusted odds ratio (aOR) of 3.99) and with low/moderate (vs. high) desire to quit vaping (aOR of 2.78). Overall, these data do not provide information concerning TFNPs per se, as the study assessed ONDS as a category and not TFNPs specifically.

Differences in intention to use flavoured ONDS, including TFNPs, were compared by Leventhal et al. [73] among 1,385 EVP users and non‑users, who were current non‑users of ONDS, using data also collected in the Southern Californian youth survey study. After viewing randomised digital images of various flavoured medicinal nicotine gums, non‑medicinal nicotine gums, nicotine gummies, non‑medicinal nicotine lozenges, and TFNPs, participants were asked to rate their intention to use with responses ranging from 0 to 100. Participants were classified as past six‑month EVP non‑users, EVP users motivated to quit, and EVP users unmotivated to quit. Intention to use TFNPs was significantly lower among EVP non‑users than among EVP users, both with and without intentions to quit vaping, and among those EVP quit intention categories (motivated and unmotivated), use intentions did not differ. Collapsed analysis (which included all EVP user groups) demonstrated that the intentions to use TFNPs were lower than intentions to use NRT gum, though this effect was not statistically significant.

While the Southern Californian adolescents survey study has generated a wealth of information concerning TFNP perceptions, intentions to use, and actual use, among youth, it is geographically limited and perhaps not reflective of the broader picture in the US and elsewhere. Other studies, however, have reviewed US national datasets to examine TFNP use among youth. One study described analyses of data from the US nationally representative annual National Youth Tobacco Survey (NYTS) [74], in which the prevalence of use of tobacco products and tobacco‑free, nicotine-containing products among youth, along with factors associated with this use, were assessed [75]. In 2021 when TFNP use prevalence was first assessed by the NYTS, these analyses demonstrated that an extrapolated 6.6 million (24.1%) of US middle and high school students had ever used a tobacco product, and 3.6 million (9.3%) reported current (past 30‑day) use of any tobacco product. Among the students who reported ever use of any tobacco product, 1.9% reported ever use of TFNPs (which were classified as pouches containing nicotine powder that comes from tobacco, which users place in their mouth), and of the students who reported past 30‑day use of any tobacco product, 0.8% reported past 30‑day use of TFNPs. Among students who currently used TFNPs, 17.2% reported frequent use (on ≥20 of the past 30 days) of TFNPs and 61.6% reported use of a flavoured TFNP. Mint was the most frequently reported flavour type for TFNPs (53.5%). Overall, these data suggest that, at the time of the survey, ever use of TFNPs among US middle and high school students was low, while current and frequent use were extremely low. The authors suggest, however, that these data may act as baseline data for future monitoring of TFNP use among youth.

In a further analysis of 2021 NYTS data, Speciale et al. [76] assessed the prevalence of alternative nicotine product use among middle and high school students. While data specifically for TFNPs were not provided (TFNPs and dissolvable tobacco products were collapsed into a single ONDS category), when students were classified by cigarette use and/or ONDS usage, 187 students (approximately 1% of the survey population) had dual‑used both cigarettes and oral nicotine, and 165 students had ever used only ONDS. When students were classified by EVP use and/or oral nicotine product usage, 265 students (<2% of the population) reported using both EVPs and ONDS, and 87 (approximately 0.5%) of students reported they ever used only ONDS. ONDS ever‑use was more common among males, and ever‑use prevalence increased with age. Almost three‑quarters of those reporting ever use of ONDS were non‑Hispanic white students. Approximately half of students who had ever used ONDS had also smoked cigarettes, and approximately three‑quarters had ever used EVPs. These latter findings align with risk perception data since ever‑users of oral nicotine products generally perceived lower degrees of harm from smoking and EVP use than never‑users. In sum, these findings [76] are in general agreement with those of Gentzke et al. [75] in that the prevalence of use of TFNPs by US students in 2021 was very low.

Using data from the Altria Client Services Underage Tobacco Use Survey (UTUS), which is similar to the NYTS assesses tobacco product use in the US nationally representative samples of individuals aged 13‑20, Cheng et al. [77] reported that, between 2020 and 2022, approximately 40% of adolescents and half of the young adults were aware of ONDS as a broad category. In agreement with the findings from NYTS analyses [75,76], current (past 30‑day) ONDS use was very low at less than 2% [77]. In further agreement with these studies, analyses of data from another US national sample of adolescents and young adults (aged 14‑20) who reported having ever used EVPs at least three times in their lives [78] showed that approximately a quarter of the 2,253 participants reported ever‑use of TFNPs and that frequent, current use was only seen in less than 2% of the survey sample. As previously reported from other studies, the likelihood of TFNP ever‑use and current use was dependent on age, higher among males compared with females, and higher among current smokers compared with non‑smokers. In contrast to the findings of Speciale et al. [76], however, both the ever use and current use of TFNPs were seen uniformly across all the race/ethnicity subgroups assessed [78].

One final nationally representative US survey collected data from adolescents and young adults (aged 15‑24) between 2020 and 2022 [79]. While ever use of TFNPs was reportedly low, there did appear to be an increase in ever‑use over time, from 3% in September 2020 to 5% in May 2022, with the majority of that increase seen in the period between October 2021 and May 2022. It is notable, however, that this time‑dependent increase in ever use of TFNPs does not appear to have translated into a higher prevalence of current TFNP use in the survey sample. As for other studies, ever and current use of TFNPs was associated with being older, being male, and having ever used cigarettes or EVPs and, to a lesser extent, chewing tobacco, but not with having ever used snus. Interestingly, while ever use of TFNPs was predominantly prevalent among the non‑Hispanic white population, which concurs with the findings of Speciale et al. [76], current use was evenly distributed among the non‑Hispanic white population and Hispanic populations.

In a population geographically distinct from the US population, Havermans et al. [27] investigated awareness, use, and perceptions of cigarillos, HTPs, and TFNPs, among a representative sample of the Dutch population. Participants were presented with names and typical images of the tobacco and nicotine products assessed. In general, lower awareness of TFNPs compared with cigarillos and HTPs was reported. In the adolescent cohort aged 13-17, awareness of TFNPs was approximately 9%, ever use was 0.9%, and current use was zero. Findings were similar among young adults aged 18-24. Interestingly, both awareness and ever use were slightly higher among non‑Western immigrants compared with Dutch and Western immigrants, though these are for the whole population and not just adolescents/young adults. Awareness (among the whole survey population) was the highest among those knowing other people who use it, and the use was most commonly with friends/at parties and for a reason of being out of curiosity. Among those not having ever used TFNPs, the main reason given was "unhealthy". Perceptions of harmfulness and addictiveness were slightly lower on average for TFNPs compared with combustible tobacco products and HTPs, regardless of use status. Overall, like studies in other countries, this study suggested an extremely low prevalence of use of TFNPs among Dutch youth. Similar to the Netherlands, a study of 9,515 Swiss secondary school students aged between 15 and 21, conducted in late 2021/early 2022, reported a low prevalence of current infrequent TFNP use and a very low prevalence of frequent (daily) use of TFNPs [80]. Overall, these studies indicated that the use of TFNPs was less prevalent than tobacco‑containing products, such as cigarettes, snus, snuff, and hookah, which are reported to contain more toxicants than those without tobacco and are, therefore, likely to be more harmful.

One final paper identified in the literature search concerning adolescent and young adult use patterns, and the likelihood of buying and liking TFNPs [26], is discussed later in this review. To avoid duplication, the findings will not be discussed in this section.

Overall, data from youth surveys assessed in both the US and the Netherlands suggest a low prevalence of use and interest in using TFNPs, both absolutely and relative to other tobacco and nicotine products. While this is encouraging, further assessment, including over time and in other countries in which TFNPs are marketed, is warranted to maintain awareness of the use of TFNPs among youth and to ensure that their interest in using these products remains low. This is particularly important since, for example, youth use of other alternative nicotine products such as EVPs rose sharply in a short space of time (between 2011 and 2015) in the US [81]. Although the greater proportion of youth EVP use tends to be among those with a history of use of other tobacco/nicotine products, maintaining an awareness of TFNP use among youth, and their prior product use history, is warranted to continue to estimate the overall population impact. Additionally, differential use of TFNPs among those of different race/ethnicity backgrounds requires further assessment and monitoring in order to facilitate an understanding of the THR potential of TFNPs among all segments of populations and especially those at higher risk of initiating tobacco/nicotine use.

Perceptions, Motivations, Intentions, and Actual Use of TFNPs Among Adults

Between November 2017 and February 2018, two independent surveys were conducted to both assess the overall appeal and future intention to buy ZYN TFNPs among 5,179 ZYN‑naïve consumers and to describe the demographics and patterns of use among 1,266 ZYN users [67]. Regarding appeal, ZYN appealed to a minority (11%‑12%) of never and former tobacco users. In contrast, the product was moderately to extremely appealing to 36% of current smokers, 52% of current smokeless tobacco users, and 75% of dual cigarette/smokeless tobacco users. A minority of never and former users (2% and 3%, respectively) were likely to buy ZYN, compared with 64% of dual users, 44% of current smokeless tobacco users, and 27% of current smokers. Exclusive smokeless tobacco users were most likely to buy ZYN (41%) and had little or no interest in EVPs (2%), NRT (8%), or cigarettes (2%). Regarding tobacco use status, 43% of ZYN users were former tobacco users, and among these, 13% were former cigarette smokers, 31% were former smokeless tobacco users, 53% were former cigarette and smokeless tobacco users, and 2% were former EVP and other combustible tobacco product users. Approximately a quarter were current smokeless tobacco users (26%), and 8% were current smokers. Approximately 90% of ZYN users used the product every day, and 6 mg was the most commonly reported per-pouch nicotine strength used. Over 60% of current smokers stated that they used ZYN to help reduce and/or quit cigarette smoking, and 60% of former users reported that they used ZYN to help quit other tobacco product use. Among all participants, the most popular other reason for use was that ZYN poses less risk to their health than other tobacco products, excluding cigarettes (62%), ease of use (53%), and less harmful to their health than cigarettes (49%). These data suggest that non‑users of tobacco had very little interest in ZYN. Furthermore, smokeless tobacco users were not only more interested and likely to buy ZYN compared with other tobacco users, but they represented the majority of regular ZYN users. Additionally, dual use with other products was low. Overall, the findings suggest that ZYN may help users in transitioning away from potentially more harmful forms of tobacco/nicotine use though this would need to be validated through a longitudinal assessment.

Analysing data collected from September to December 2020 from a cohort of young adults (n = 1,167; aged 19-23 years) who were earlier participants in the Southern Californian Adolescent survey, Vogel et al. [82] assessed differences in tobacco non-user perceptions and willingness to use TFNP. Participants were shown images of TFNPs, after which they responded to questions regarding perceptions and intentions to use TFNPs and their hypothetical choice of cigarettes or EVPs over TFNPs. Data were analysed in sub‑cohorts according to their tobacco/nicotine use status. Overall awareness of TFNPs before the study was low, averaging approximately 11% in the whole cohort and ranging from 8.8% among tobacco non‑users to 19.7% among dual users of combustible and non‑combustible tobacco products. Willingness to use TFNPs was significantly higher among non‑combustible-only users (33.8%), combustible-only users (29.3%), and dual users (43.9%), compared with non‑users (14.7%), noting that "probably not" responses were coded as an affirmation of willingness. Among these tobacco non‑users, approximately 85% of participants were "definitely not" willing to use TFNPs. Overall, approximately half of the participants were uncertain whether TFNPs were less harmful than cigarettes, a similar proportion were uncertain whether pouches were less harmful than EVPs, and perceptions did not differ across tobacco use status groups. Hypothetical choice of EVPs over TFNPs aligned with participants’ tobacco use status such that those using EVPs or other non‑combustible products (either alone or as part of dual use with combustible tobacco) had greater odds than non‑users of reporting that they would use EVPs over TFNPs, but exclusive combustible tobacco users and tobacco non‑users did not differ in this outcome. In contrast, all tobacco product use groups were more likely than non‑users to choose cigarettes over TFNPs.

These findings are supported by a further similar study that assessed awareness, interest, and ever use of TFNPs among a nationally representative sample of adult smokers in the US [83]. In this study, data were collected using a web‑based survey between 21 January and 4 February 2021 among 1,018 current established smokers who were 18 years of age or older. Approximately one‑third of participants were aware of TFNPs, and 5.5% of all smokers had ever tried using them. Awareness of TFNPs was significantly higher among those who had ever used smokeless tobacco products (aOR of 3.38) and significantly higher among lower‑age participants. Compared with those aged ≥45 years, individuals aged 18‑44 years were approximately three times more likely to have ever used TFNPs. Reported use was significantly higher among individuals who had ever attempted to quit smoking using traditional methods, such as medication, counseling, or other supporting programmes (aOR of 4.18), and among those who had ever used smokeless tobacco products (aOR of 10.0). Additionally, significantly increased odds of having an interest in using TFNPs were found among those having plans to quit less than six months in the future (aOR of 1.90), having a prior quit attempt using traditional methods (aOR of 1.62) or another tobacco product (aOR of 2.08), and having ever used TFNPs (aOR of 5.82).

In a further US study, Sparrock et al. [84] gathered data on awareness, beliefs, susceptibility, and the use of TFNPs among adult (aged 18+) current users of tobacco products, including cigarettes, cigars, EVPs, hookah, other combustible tobacco products, and smokeless tobacco products, in 2021. This is an important area since the main principle of THR recognises to maximise the population health impact of a reduced-risk product, and it must be used by sufficient numbers of current users of more harmful tobacco products and support switching. Participants were presented with images of popular brands of TFNPs, such as Dryft, on!, Velo, and Zyn, and asked questions regarding awareness, ever and current use, and curiosity. Awareness, ever use, and current use were the greatest among those aged 18‑30 and 31‑45. For current use, those in these age groups were approximately 55-60% more likely to be currently using TFNPs than those aged 61+. Ever and current use were associated with a higher level of education, higher income, and living in large cities, although these effects were not statistically significant. As has been reported in youth studies (e.g., [76,78,79]), current use was more common among cigarette smokers and EVP users, as well as among users of smokeless tobacco, but was less common among cigar and other combustible tobacco product users. Overall, 23.2% believed that TFNPs are less harmful than smokeless tobacco, 16.1% believed that TFNPs are less addictive than smokeless tobacco, 18.9% believed that using TFNPs occasionally is not harmful, 14.6% believed that occasional TFNP use is not addictive, 33.2% believed that TFNPs are socially acceptable, and 19.1% believed that TFNPs are for someone like themselves. Furthermore, holding favourable beliefs about TFNPs was associated with more advanced TFNP use statuses; for example, those who agreed that TFNPs were less harmful than smokeless tobacco were more likely to be susceptible never, ever‑not‑current, and current TFNP users. Additionally, those who were unsure about the potential harms and addictiveness of TFNPs had lower odds than ever‑but‑not‑current TFNP users and current TFNP users; for example, those who reported “don’t know” concerning “using TFNPs occasionally is not harmful” had lower odds of being ever‑but‑not‑current TFNP users and current TFNP users. These data are informative since, as we have observed for other nicotine products such as EVPs [85], misperceptions of the relative risk of TFNPs compared with more harmful tobacco products may be a barrier to switching. Promisingly though, the findings of Sparrock et al. [84], when taken alongside the findings of other prior studies [83,86], suggest that awareness of, experimentation with, and current use of, TFNPs substantially increased between 2020 and 2021 among tobacco users.

The prevalence of TFNP use among adults in Great Britain was estimated by Tattan‑Birch et al. [87], in a study that assessed how use differs by age group, gender, social grade, smoking status, and the use of other nicotine‑containing products. Data from the Smoking Toolkit Study [88], a monthly cross‑sectional survey that recruits a representative sample of adults (≥18 years of age) in Great Britain, were analysed. A total of 25,698 participants were surveyed between November 2020 and October 2021 and asked whether they currently use TFNPs. The use of TFNPs at the time of the survey was rare, with a weighted, estimated prevalence of current TFNP use of 0.26%. The prevalence of TFNP use increased from 0.14% in November 2020 to 0.32% in October 2021, with men significantly and almost four times more likely to report use of TFNPs (0.42%) compared with women (0.09%). The use of TFNPs was significantly higher among those aged 18-24 years (0.49%) compared with those aged 55-64 (0.15%) and 65 or over (0.06%). Relevantly, the authors noted that long‑term former smokers (0.24%), recent former smokers (0.97%), and current smokers (0.87%) all reported significantly higher prevalence of TFNP use compared with never smokers (0.06%). EVP users were significantly more likely to report TFNP use than non‑EVP users (1.64% and 0.15%, respectively), and NRT users were significantly more likely to report TFNP use than non‑NRT users (2.02% and 0.21%, respectively). Overall, these data suggest a predominance of TFNP use among those with a history of use, or are current users, of tobacco and nicotine products.

Awareness, ever use, and current use, of TFNPs were assessed by Brose et al. [89] in an examination of self‑reported cross‑sectional data from a 2019 wave of a UK longitudinal online survey of 3,883 adults who were either current smokers, former smokers, and/or vapers. Approximately 16% of the survey population had awareness of TFNPs, and fewer (3.1%) had ever seen TFNPs for sale in shops or online. Ever use of TFNPs was observed in 4.4% of participants, while 2.7% were current TFNP users. Men were reportedly more likely to have used TFNPs than women, a finding which is in accordance with a similar study by Tattan‑Birch et al. [87]. Groups more likely to have ever used were those aged under 45, those with a university education, and those from London. Those currently vaping and smoking were more likely to have ever used TFNPs than those who were exclusively smoking, and those currently not smoking or vaping, and those exclusively vaping, had similar levels of ever use as those exclusively smoking. In their concluding remarks, the authors suggested that the low level of use is unlikely to substantially reduce the public health impact of smoking, which is aligned with the concept that, for a novel tobacco product to play a role in population‑level THR, it must not only be reduced risk but also used as a smoking substitute. In contrast, Shao et al. [90], in their evaluation of public perceptions and discussions among a total of 2,410 posts related to TFNPs on the internet platform “Reddit”, suggested a growing popularity of the TFNP category. Their finding of a clear upward trend in the number of posts related to TFNPs, with the most significant rise occurring between February 2019 (two posts) to November 2020 (54 posts), suggests that the THR potential of TFNPs may be increasing, particularly since over two‑thirds of posts were related to quitting. However, whether this relates to quitting smoking, vaping, TFNPs, or all nicotine use, is unclear.

In a cross‑sectional analysis of 2,507 US participants in the International Tobacco Control Four Country Smoking and Vaping Survey, a population‑based survey of current and former cigarette smokers and nicotine vaping product users in four countries, including the US [91], Felicione et al. [86] assessed prevalence and correlates of ONDS awareness and use, noting that data were provided for the ONDS category and not specifically for TFNPs, between February and June 2020. The study found that, while almost 20% of respondents had heard of ONDS, a value that concurs with other studies described above, only 3% reported ever use, and an even smaller proportion (0.9%) were current TFNP users. Ever use of TFNPs was more common among younger adults (aged 18-24 years), males, and current users of smokeless tobacco products. Importantly, ONDS use prevalence was higher among those who reported having made attempts to stop smoking or vaping.

The effect of features on the packaging of TFNPs on risk perceptions has been examined among adult tobacco users and non‑tobacco users [92] in a study that used a convenience sample of 301 such individuals in the US in 2021. In this study, participants viewed online images of a random sample of TFNP packaging with different flavour descriptors, nicotine strengths, and addiction warning labels and responded to questions concerning the substitutability of TFNPs for cigarettes and smokeless tobacco. Interestingly, the study found a significant effect on nicotine strength. Compared with the pack images that did not display nicotine strength, participants who viewed the pack images, displaying a 3 mg nicotine strength, reported a lower perceived substitutability for cigarettes. Compared with non‑users, most tobacco user groups reported significantly lower perceived harm and addictiveness. Regarding nicotine strength, participants viewing the pack images displaying a 6 mg nicotine strength reported significantly lower perceived harm compared with packs that did not display nicotine strength. Participants viewing the pack images displaying a 6 mg nicotine strength also reported significantly lower perceived addictiveness compared with the pack that did not display the nicotine concentration. Regarding perceptions, all tobacco user groups perceived a lower risk of both harm to health and addiction compared with non‑users of tobacco, an effect that is also seen for other reduced-risk tobacco products [93].

Data from a prospective observational study conducted among 100 healthy US adults aged 21 years old or older were described by Campbell et al. [94]. Participants were daily menthol and/or non‑menthol cigarette smokers of at least seven cigarettes per day (CPD) who had no intention of quitting tobacco use over the eight‑week study period. The primary objective of the study was to describe the patterns of use of two variants of TFNPs (Velo with mint or citrus flavour and containing 4 mg of nicotine per pouch), and cigarette smoking, over a six‑week actual use period using self‑reported data from a daily eDiary. Reported use of TFNPs decreased over time. The proportion of participants reporting they did not use TFNPs was 0% in week one compared to 15.5% reporting non-use of TFNPs in week six. Among those who continued using the TFNPs throughout the study, the proportion of participants using between one and six pouches per day decreased between week one and week six (from 72% to 41%), while the proportion using seven or more pouches per day increased from 27% to 42% in the same timeframe. Self‑reported cigarette smoking decreased over the study period for 82 of the study participants. At week six, approximately 16% of participants reported that they had reduced their CPD by more than 50%, 18% reduced their CPD by between 30% and 50%, and almost half reduced their CPD by between 1% and 30%. Overall, the authors suggest that these trends over time are indicative of the ability of TFNPs to displace cigarette use among smokers and, therefore, suggest a potential positive role for TFNPs in THR since they present an acceptable alternative for adult smokers to replace cigarette consumption.

Using the 2020 ITC Four Country Smoking and Vaping Survey as a data source, Li et al. [95] examined patterns of the use of non‑cigarette tobacco and nicotine products, including TFNPs, among 9,112 adult (aged 18+) current smokers and recent adult former smokers (quitters). Overall and across the total survey population, TFNP use was the least reported product and was seen in only 0.8% of participants. This compares, for example, with EVP use among 14% of participants and NRT use among 11%. TFNP use was observed in similar proportions of participants in England, Canada, and the US, but was only seen in 0.1% of Australians. Current TFNP use was twice as likely overall among males compared with females and also significantly more likely among young adults (aged 18‑24) compared with older adults. When assessed by smoking status, TFNP use was significantly less likely among former smokers compared with current smokers. While this may be suggestive of the dual use of cigarettes and TFNPs, it is noteworthy that the actual percentages were very small (only 1% of current smokers were TFNP dual users). It is also notable that the overwhelming majority of smokers (74%) did not use any other nicotine‑containing product. As for other areas of interest such as potential youth use, patterns of TFNP use among adult smokers warrants continued oversight since it has implications for the population health impact of TFNPs and for the ability of these products to support both smokers in switching away from cigarettes and THR.

Morean et al. [96] examined awareness of, susceptibility to, and use of, TFNPs among 609 young adults aged 18‑25 using an online convenience survey sample in the US. Comparative risk perceptions relative to smokeless tobacco were also assessed. The prevalence of ever use of TFNPs (10.3%) was lower than that for all other tobacco products, which ranged from approximately 14% for smokeless tobacco to approximately 75% for EVPs. Approximately 42% of participants had heard of TFNPs and 23.5% were deemed by the authors as susceptible to future use. Individuals who were susceptible to future TFNP use or had ever used TFNPs were significantly more likely to perceive lower degrees of risks to the health of using TFNPs compared with using smokeless tobacco across various health effects domains and to perceive a lower degree of addiction risk, compared with those who were non‑susceptible. Overall, these findings are encouraging, firstly since TFNP use prevalence was lower than that of other nicotine/tobacco products in this user age group, which can act as a proxy for youth, and secondly since TFNP use is more likely to be observed among those with a perception of lower relative risk of TFNPs compared with smokeless tobacco.

One final paper identified in the literature search estimated use patterns and the likelihood of buying and liking oral nicotine products, including ZYN TFNPs among 6,131 US adolescents, youth, and young adults, in 2021 [26]. Assessing the self‑reported use of TFNPs, toothpicks, gums, lozenges, and tablets, a randomly selected subset of participants viewed images of ZYN TFNPs and "Lucy" nicotine gum marketing materials and responded to questions in domains such as liking and intentions to buy. Among the 2,468 adults aged 21‑40 in the survey population, ever use of oral nicotine products was seen among approximately 46% of participants, while past 30‑day and past seven‑day use was observed among approximately 29% and 24%, respectively. Notably, while no statistical analyses were performed on these actual use data, this prevalence of use estimates appears substantially higher than those in the younger, aged 13‑20, population. For the past 30‑day use of TFNPs among adults, the use of sweet/dessert flavours was more common than the use of mint/menthol flavours, while the prevalence of use of tobacco‑flavoured TFNPs was among the lowest for all the flavours assessed. These patterns were similar among the younger cohort. Regarding liking ZYN TFNPs based on advertising, the average score based on a 5‑point Likert scale, which ranged from "Not at all" to "Extremely", was 1.03 among adult never users and 2.09 among adult ever users. It is worth noting that it was not clear what the participants were never and ever users of (i.e., TFNPs specifically or all oral nicotine products), which limits any analysis that can be made. Although no statistical comparisons were made, these values appeared substantially higher than those observed among the younger participants (0.61 and 1.33, respectively). There was some evidence that TFNP flavours may be appealing to the younger cohort, as participants aged 13-20 were significantly more likely to "intend to buy ZYN TFNPs" if they perceived a product was marketed as having good-tasting flavours. Again, no between-age group comparisons were made, and, while this flavour appeal was not significant in adults, the odds ratio was only marginally lower than that observed for the younger cohort. Finally, regarding the likelihood of buying ZYN TFNPs, the average score, based on a similar 5‑point Likert scale to the liking data, was 1.08 among adult never users and 2.44 among adult ever users. In the younger cohort, these values were again lower at 0.65 and 0.60, respectively. In summary, these data appear to suggest that adolescent and young adult appeal and the likelihood of use of TFNPs is lower than that observed among adults. However, the study only assessed one brand of TFNP, so it is difficult to extrapolate the findings to the broader product category. Future studies could perhaps expose participants to a range of marketing materials from different manufacturers and examine intentions to use and actual use.

In summary regarding adult use of TFNPs, the studies assessed in this review generally show that use prevalence is low, though perhaps growing as awareness of TFNPs develops over time. Interestingly, comparing different studies suggests that there may be regional and/or cultural differences in the use of TFNPs. In terms of the potential role of TFNPs in THR, there are two important considerations emerging from the studies examined in this review. Firstly, adult appeal and use of TFNPs appear to be predominant among those with a history of tobacco/nicotine use, particularly among smokers and users of smokeless tobacco products (i.e., snus). Secondly, the prevalence of TFNP use among adults appears to significantly outweigh the use of TFNPs among youth and young adults. Taken together, these findings are indicative of a THR potential of TFNPs, which is not mitigated to any significant degree by use among unintended populations. It is also apparent from the literature that there is a good understanding of the relative risks of TFNP use compared with the use of other tobacco/nicotine products. This can support the THR potential of TFNPs since risk perceptions are understood to be a driver of intentions to use and actual use. For other potentially reduced-risk nicotine products such as EVPs, negative risk perceptions have been described as a barrier to uptake by smokers [85]. This does not appear to be the case, at least at present, for TFNPs.

There is some evidence of the "switching" potential of TFNPs, with some data alluding to the ability of TFNPs to reduce, or act as a complete substitute for, cigarette smoking. While this is promising, given that switching would reduce exposure to chemical toxicants, as outlined earlier in this review, the evidence for assisting switching is not universal, and the topic has not been widely examined. This suggests that further studies are required to determine whether TFNP use can indeed act as a substitute for cigarette smoking and under which conditions. Other, additional, studies are also required in order to monitor the TFNP use landscape, to ensure that use among youth and non‑users of nicotine remains low, and to assess whether the THR potential of TFNPs is increasing due to greater uptake by adult smokers. Furthermore, since there may be differences between different products within the TFNP category in terms of appeal and actual use, further studies may be needed to assess individual products, and not just the TFNP category as a whole, in order to generate an accurate reflection of their THR potential.

Emerging Topics on Perceptions of TFNPs

The use of products containing synthetic nicotine is an emerging area of research as manufacturers have begun utilising nicotine not sourced from tobacco and free of tobacco-derived components [97]. Two studies identified in this section explored the influence of the description of synthetic "tobacco-free" nicotine on product perceptions for TFNP users and non-users. Morean et al. [98] examined 630 US young adults (aged 18-25 years) perceptions of synthetic TFN versus tobacco‑derived nicotine (TDN) pouches. The authors examined associations between product perceptions, product awareness, and use. Participants were informed whether the nicotine in each product was derived, either synthetically or from tobacco. Approximately one‑third of participants were aware of it, 29.2% were susceptible to it, and 3.8% had used TFNPs. Awareness, susceptibility, and use were all reported to be disproportionately associated with perceiving TFN pouches as "less harmful" or otherwise "better" than TDN pouches. For example, those who were susceptible to TFNP use held stronger perceptions that TFNPs are less harmful to a person’s health, less harmful to a person’s heart, less addictive, less expensive, and less harmful to a person’s mouth or gums than those participants who had never used and were not susceptible to TFNPs. Thus, the use of "tobacco‑free" descriptors may impact risk perceptions and the appeal of nicotine pouches among young adults, and this, in turn, may increase susceptibility to future use. The interpretation of a tobacco‑free descriptor, and what it means for perceptions of product constituents, is also of importance. Morean et al. [99] assessed such interpretation among 2,464 young adults, who reported whether cigarettes, smokeless tobacco products, EVPs, and TFNPs contain nicotine that comes from tobacco always, sometimes, or never. Interestingly, while the majority (57.8%) of participants accurately interpreted TFN to mean containing nicotine but no tobacco, others misinterpreted TFN products as containing tobacco only (10.8%), both nicotine and tobacco (14.1%), or neither nicotine nor tobacco (17.1%).

A further study evaluated whether exposure to “tobacco-free” warning labels such as those found on products like the "Puff Bar" EVP and "FRĒ" TFNPs was associated with differing perceptions of these products [100]. The study included 239 young males, at least 18 years old (average 20 years old), who were enrolled in a cohort study and invited to take part in a brief online survey. Among participants, over 20% reported using tobacco or nicotine products in the past 30 days, including 13.7% who used EVPs and 2.6% who used TFNPs in the last 30 days. For FRĒ TFNP, viewing the package with the “non‑tobacco” label (as opposed to the standard FDA nicotine warning) was associated with being more likely to perceive nicotine pouches as less harmful than smokeless tobacco. This led the authors to suggest that the “non‑tobacco” qualifier in the FRĒ warning label helped participants correctly place TFNPs at the reduced‑harm end on a relative risk scale. Taking this view into consideration, along with the studies described above, what is perhaps needed is a better understanding of the perceptions of TFNPs and how these depend on how they are marketed and what descriptors are used on the packaging. Given that perceptions may influence intentions to use and future actual use of TFNPs, assessing perceptions among both adult smokers who may switch to using TFNPs for the benefit of their health, as well as the perceptions among nicotine‑naïve individuals including youth, is necessary to inform an overall population‑level health impact assessment.

Summary and suggestions for future research

THR refers to strategies designed to reduce the health risks associated with tobacco smoking, but which may involve continued use of nicotine/ tobacco. NGPs, such as electronic vapour products, heated tobacco products, and oral nicotine delivery systems, eliminate the process of tobacco combustion, meaning they contain and produce fewer and significantly lower levels of harmful chemicals compared to cigarette smoke. Such products have the potential to offer a reduced-risk alternative compared to smoking cigarettes.

In addition to being less harmful than cigarettes, to fulfill their THR potential on a population level, novel nicotine or tobacco products must also be acceptable for adult smokers and, importantly, not attract unintended populations, such as never-smokers, ex-smokers, or youth. Given this, any examination of the THR potential of a novel nicotine product should also take these factors into account.

There is a significant body of literature, including long-term population studies, examining the reduced risk potential of snus, an oral smokeless tobacco product primarily used in Sweden and Norway [43]. TFNPs are a relatively new and emerging category of oral nicotine products. The body scientific of literature relating to the TFNP product category has grown over recent years, and the aim of this review was to collate and critically examine this scientific literature in order to understand whether TFNPs have THR potential. This involved assessment of whether they had the potential to reduce the health risks associated with smoking, whether smokers find them an acceptable alternative to smoking cigarettes, and whether they were used by or attractive to any non-intended user groups (e.g., non-smokers or youth).

Much of the scientific research presented in this review supports that TFNPs present a reduced exposure risk at an individual level. As TFNPs neither contain nor burn tobacco, extracts of TFNPs contain fewer and significantly lower levels of identified chemical toxicants than cigarette smoke extracts. In addition, some *in vitro* studies, exploring whether the reduced levels of harmful chemicals observed in the product translate to reduced biological responses in both cellular and tissue models, have demonstrated that toxicant exposure may be comparable to exposure associated with NRT use [28,32]. In a user switching from cigarette smoking, this reduction in exposure to harmful chemicals may lead to a reduced risk of harm to health, and evidence of such a health risk reduction was described in a biomarker study assessing various indicators of disease likelihood [57]. In addition, the literature generally supports that TFNPs convey a reduced toxicological risk compared with cigarettes and snus. In their totality, these studies support that TFNPs can reduce disease risk at the level of the individual. Potentially lower-risk nicotine products such as TFNPs can only achieve their THR potential and be of population health benefit if adult smokers use the products instead of cigarettes, but, importantly, they are not used by unintended populations, such as never-smokers, ex-smokers, or youth. Some evidence supports that TFNPs can facilitate either partial or complete switching, though the data provided in such studies are currently limited. Some studies have assessed the abuse liability or addiction potential of TFNPs, and such assessments are generally thought to be informative to an estimation of the likelihood that smokers will find TFNPs to be a viable alternative to cigarette smoking. While peak blood nicotine levels among users of some TFNPs may reach those approximating the levels seen in cigarette smokers, the time course is much slower due to the gingival, and not lung, route of nicotine absorption. While this suggests a lower abuse liability than cigarettes, it does indicate that TFNPs have at least some degree of abuse liability, and this may be important to smokers’ uptake of TFNP. In addition, subjects in clinical studies using some TFNPs appear to report satisfaction, reductions in negative effects associated with smoking abstention such as withdrawal symptoms and craving, and relief. These findings suggest that TFNPs can act as acceptable alternatives to cigarette smoking, and this is supported by data suggesting that TFNPs can help smokers switch away from cigarette smoking. With the increasing availability of TFNP products on markets, this ability needs to be assessed over time. Furthermore, longitudinal assessments of behaviour change over prolonged periods of time, which have not been reported in the literature to date, could facilitate an understanding of the substitutability of cigarettes for TFNPs among smokers.

The potentially beneficial impacts of TFNP availability on population health could be mitigated by use among unintended populations, such as non‑users of nicotine and youth/young adults. At present, however, the indications are that TFNPs predominantly appeal to, and are used by, existing users of combustible and non‑combustible tobacco products. In addition, some data suggest that appeal, curiosity, and prevalence of use are higher among adults than youth/young adults. Both of these are positive findings, and should the situation remain the same, any deleterious impacts on population health should be minimised. However, this situation needs to be carefully monitored, due to the growing availability, awareness, and use of TFNPs, which has been reported in several papers and likely reflects the growing TFNP market. Such monitoring and future surveillance are important as they will facilitate an ongoing assessment of the population health potential of TFNPs, as well as allow mitigating actions to be taken should unintended use become detrimental to population health. Given that some manufacturers have switched to using synthetic nicotine, and as adult consumers have also increased their preference for nicotine not sourced from tobacco and free of tobacco-derived components, monitoring should include ongoing assessment of how the use of descriptors on product packaging and marketing materials, such as "tobacco-free" or "non‑tobacco", may influence curiosity, intentions to use, and actual use. Whether manufactured naturally or synthetically, the nicotine molecule has the same chemical formula, though we do not yet have a full understanding of the pharmacological and behavioural properties of the R and S enantiomers of nicotine, which exist in different proportions in TDN and synthetic nicotine. However, differences exist in the way that TDN and synthetic nicotine are isolated and/or produced, leading to potential reductions in impurities in synthetic compared to TDN [97].

## Conclusions

Based on the reviewed evidence, TFNPs contain significantly fewer and lower levels of harmful chemicals, have a reduced toxicological impact compared to cigarette smoke, and may convey lower health risks compared to smoking. The population health benefit appears positive; however, the apparent lack of appeal to nicotine non‑users and youth needs to be monitored to maintain vigilance and further assess the population health benefit. Additionally, TFNP regulation must consider both the potential benefits for adult smokers in aiding switching away from smoking and any detrimental effects of unintended use.
